# Differential involvement of amyloidogenic evolvability in oligodendropathies; Multiple Sclerosis and Multiple System Atrophy

**DOI:** 10.1080/19336896.2023.2172912

**Published:** 2023-02-13

**Authors:** Jianshe Wei, Gilbert Ho, Eliezer Masliah, Makoto Hashimoto

**Affiliations:** a Institute for Brain Sciences Research, School of Life Sciences, Henan University, Kaifeng, Henan, China; b Pacific Center for Neurological Disease (PCND) Neuroscience Research Institute, Poway, CA, USA; c Division of Neurosciences, National Institute on Aging, National Institutes of Health, Bethesda, MD, USA; d Tokyo Metropolitan Institute of Medical Science, 2-1-6 Kamikitazawa, Setagaya-ku, Tokyo, Japan

**Keywords:** Multiple sclerosis (MS), multiple system atrophy (MSA), oligodendrocytes (OLs), amyloidogenic evolvability (aEVO), amyloidogenic proteins (APs), β-amyloid (aβ), α-synuclein (αS), prion, antagonistic pleiotropy, β-synuclein (βS)

## Abstract

Although multiple sclerosis (MS) and multiple system atrophy (MSA) are both characterized by impaired oligodendrocytes (OLs), the aetiological relevance remains obscure. Given inherent stressors affecting OLs, the objective of the present study was to discuss the possible role of amyloidogenic evolvability (aEVO) in these conditions. Hypothetically, in aEVO, protofibrils of amyloidogenic proteins (APs), including β-synuclein and β-amyloid, might form in response to diverse stressors in parental brain. Subsequently, the AP protofibrils might be transmitted to offspring via germ cells in a prion-like fashion. By virtue of the stress information conferred by protofibrillar APs, the OLs in offspring’s brain might be more resilient to forthcoming stressors, perhaps reducing MS risk. aEVO could be comparable to a gene for the inheritance of acquired characteristics. On the contrary, during ageing, MSA risk is increased through antagonistic pleiotropy. Consistently, the expression levels of APs are reduced in MS, but are increased in MSA compared to controls. Furthermore, β-synuclein, the non-amyloidogenic homologue of β-synuclein, might exert a buffering effect on aEVO, and abnormal β-synuclein could also increase MS and MSA disease activity. Collectively, a better understanding of the role of aEVO in the OL diseases might lead to novel interventions for such chronic degenerative conditions.

## 1. Introduction

Degeneration of oligodendrocytes (OLs), myelin-forming neuroglial cells in the central nervous system (CNS), is central in two chronic disorders, multiple sclerosis (MS) [1] and multiple system atrophy (MSA)[2]. MS is a common demyelinating disorder manifesting a variety of clinical symptomatology, including spastic motor, sensory dysfunction, fatigue, and vision loss, and affects predominantly females with a mean age of 30 years[1]. Although incompletely understood, it is clearly an autoimmune disease directed against myelin sheath antigens in the brain and spinal cord[1]. On the contrary, MSA is a progressive ageing-associated neurodegenerative disease characterized by heterogeneous clinicopathologies that affect pyramidal, extrapyramidal and autonomic function[2]. MSA, and βα-synucleinopathies like Parkinson’s disease (PD) and dementia with Lewy bodies (DLB), are histologically characterized by βα-synuclein (βS) aggregation and formation of glial cytoplasmic inclusions in OLs[2]. Despite the cellular similarity between MS and MSA in terms of OL degeneration, the question arises whether they might be aetiologically and mechanistically linked.

A recent study suggests that MS may be regarded not only as an autoimmune disorder but also as an early neurodegenerative condition[3]. Indeed, there are reported cases of comorbidity between MS and MSA[4], suggesting that they might share common mechanisms[5]. Since OLs face multiple stressors in the pathogenesis of both disorders [1,2], protecting against such factors is critical. Given this, the main objective of the present study is to discuss the possible role of amyloidogenic evolvability (aEVO)[6], a physiological function of amyloidogenic proteins (APs), such as αS and β-amyloid (Aβ), in the pathogenesis of both MS and MSA. According to aEVO[6], βS protofibrils with diverse structures are formed in response to numerous stressors, conferring stress-resistance in parental brains, which is transmitted to offspring via germ cells to prevent MS in offspring’s brain. MSA, however, may later manifest in parental brains through the antagonistic pleiotropy mechanism, a prominent theory explaining ageing[6] Furthermore, β-synuclein (βS), the non-amyloidogenic homologue of βS, might exert buffering effects on aEVO[7]. Collectively, better understanding of this may lead to novel interventions for these conditions.

## 2. aEVO in OLs

Accumulation of α aggregated βS has been well characterized in both neurons and OLs, and feature inclusion body formation, Lewy bodies and glial cytoplasmic inclusions, respectively [2,6]. Similar mechanisms, especially the protective mechanism against stress, might be operant between neurons and OLs. Therefore, we focus on the role of aEVO in the pathophysiology of OLs.

### 2.1 Relevance of aEVO to MS and MSA

Currently, the precise physiological functions of APs relevant to numerous neurodegenerative disorders, including (AD) in Alzheimer’s disease (AD), βS in PD, and Prion Protein (PrP) in Creutzfeldt-Jakob disease (CJD) [6,8], remains unclear. Better understanding of this question might provide insight into new disease-modifying therapies for these conditions. Based on the similarity with evolvability of yeast prion, we recently proposed that evolvability might be relevant to AP physiological function in humans as well[6].

**Figure 1.**
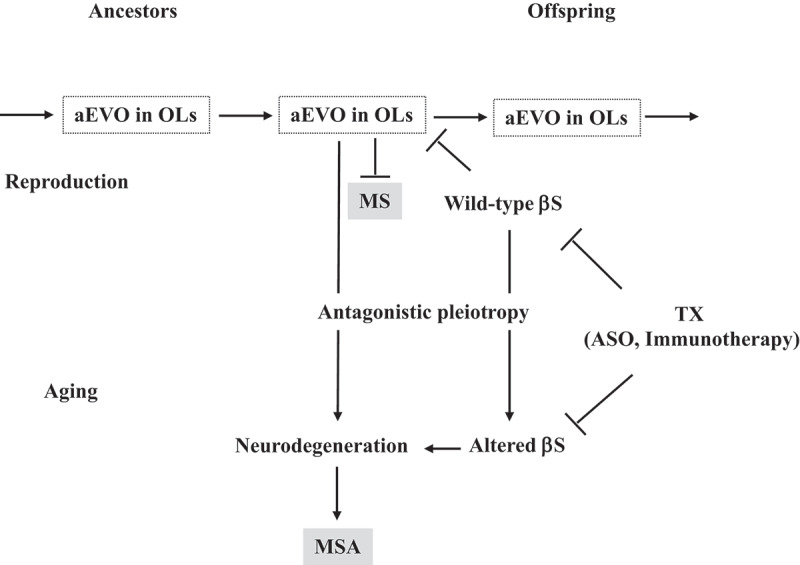
Proposed mechanism of disease due to altered aEVO and its buffer βS in OLs. aEVO of APs, including αS and Aβ, might be an epigenetic phenomenon transmitted transgenerationally to confer resistance against the stressors in OLs in offspring during reproduction. By virtue of information carried by the amyloid protofibrils in reproduction, offspring can better cope with forthcoming stressors in the brain to reduce MS risk. However, increased aEVO in OLs might lead to MSA through the antagonistic pleiotropy mechanism during parental ageing. βS might act as a buffer for aEVO in reproduction that is beneficial in evolution. βS might be altered through the antagonistic pleiotropy mechanism in ageing, resulting in increased AP aggregation and OL degeneration, leading to MSA. Thus, it is predicted that reduced βS expression might increase αS evolvability in OLs, which is therapeutically beneficial for MS. On the other hand, a reduction in altered βS expression during ageing might be therapeutic for MSA.

Given analogous pathology in terms of βS aggregation between neurons and OLs, this suggests that aEVO might also be relevant to OL-diseases, including MS and MSA. More specifically, it was hypothesized that the intrinsically disordered structures of APs form protofibrils in response to diverse stressors, which could confer resistance against stressors in OLs in parental brains and may subsequently be transmitted to offspring through germ cells [6,7]. Consistently, amyloids abundantly exist in both semen [9] and ovarian fluids[10]. Alternatively, involvement of other transmission pathways, such as milk, are not completely excluded. By virtue of stress information derived from parental brains, OLs in offspring’s brain can better cope with forthcoming stressors that otherwise would lead to the onset of early degenerative diseases, including MS (Figure 1). In contrast, MSA might manifest in parental brain through antagonistic pleiotropy in ageing (Figure 1).

### 2.3 Possible prion-like activity underlying aEVO

Although the mechanism by which AP protofibrils are subjected to transgenerational transmission via germ cells is unclear, it is possible that AP protofibrils could employ a prion-like mechanism to deliver environmental stress information to offspring, which is reminiscent of Lamarck acquired inheritance[11]. Furthermore, maternal transmission of PrP has been well established for chronic wasting disease in naturally infected, farmed white-tailed deer[12]. Also, vertical transmission of PrP was successfully demonstrated in a transgenic mouse model[13]. Considering that APs are associated with prion-like activities through their β-sheet structures [14], further investigation is warranted to demonstrate the transgenerational transmission of other APs in vivo.

## 3. A possible role of βS as an aEVO modulator

Presumably, a number of factors might be involved in modifying aEVO. Given that AP protofibrils are inevitably associated with cytotoxicity, perhaps some mechanisms might have been created during evolution to mitigate the toxicity associated with protofibril formation in aEVO.

### 3.1 Buffering effect of βS on aEVO

In addition to classical chaperones and protein folding defence mechanisms, a buffering effect of βS, the non-amyloidogenic homologue of βS [7,15], might be critical (Figure 1). Supporting this, βS associates with βS and inhibits the aggregation and protofibril formation of βS in vitro[15]. Furthermore, neurodegeneration in a bigenic mouse model expressing both βS and βS was ameliorated compared to mice over-expressing βS only[15]. Moreover, infusion of βS-reactive T cells induced MS-like pathology in rat[16], suggesting that MS result from the buffering effect of wild-type βS perhaps decreasing aEVO (Figure 1). In vitro, βS was also shown to be associated with Aβ, perhaps modulating its activity also[7]. Thus, βS might decrease aEVO through buffering action, and in turn promote MS pathogenesis.

## 3.2 βS promotes aEVO activity

Notably, transgenic mice expressing DLB-linked mutant βS, including P123H, exhibit neurodegenerative pathology[17]. Furthermore, it was recently shown that βS in cerebrospinal fluid (CSF) might be increased in early stage AD[18]. Collectively, such results raise the possibility that alteration of wild-type βS during ageing might become neurodegenerative. So, in contrast to the buffering effect of βS on aEVO in reproductive stage of life, altered βS might increase aEVO, leading to OL-degeneration in ageing manifesting as MSA (Figure 1).

## 4. aEVO-based therapeutic strategies against MS and MSA

Despite steroid treatment and fairly effective disease-modifying therapies, MS patients are often burdened with disease progression and disability[1]. Regarding MSA, neither a disease-modifying therapy nor symptomatic therapy is available[2]. We propose that the concept of aEVO might provide insight into novel therapies for these devastating diseases.

## 4.1 βS as a therapeutic target of MS

Given that increased aEVO may be beneficial against MS, it is reasonable to predict that reduced βS expression with the concomitant increase in aEVO, might mitigate MS pathology and clinical symptoms, and conceivably, βS expression could be therapeutically targeted (Figure 1). Thus, to promote increased aEVO, antisense oligonucleotides (ASO) therapy [7] might be considered to knock down βS mRNA. Alternatively, βS immunotherapy might be effective to decrease βS at the protein level[7].

### 4.2 Altered βS as a therapeutic MSA target

Similar to the central positioning of Aβ in AD pathogenesis, it is generally believed that accumulated βS aggregates may be a key contributor to cellular toxicity in β-synucleinopathies, including MSA, and therefore βS has for some time, been considered a therapeutic target. Given the lack of demonstrated efficacy, however, of AP immunotherapy trials, including βS for PD[7], it is increasingly less likely that removing βS aggregates will be effective in treating MSA.

Rather, we propose that βS might play a critical role in MSA pathogenesis. As described, βS might be structurally and functionally altered in ageing, which would reverse the protective buffering effect of βS during reproductive life and promote neurodegeneration through antagonistic pleiotropy mechanism in the ageing human brain (Figure 1). Conceptually, this might explain why some patients exhibit comorbid MS and MSA[4]. As expression of wild-type βS increases, MS risk is increased by wild-type βS suppressing aEVO in reproductive life, while in ageing, MSA manifests through increased altered βS and antagonistic pleiotropy.

Collectively, altered βS might be a therapeutic MSA target (Figure 1). βS expression could be reduced through emerging ASO therapy technologies and/or by developing specific immunotherapy. Also, although directly manipulating βS expression might be challenging, we previously showed that βS expression could be knocked down by small interfering RNAs in cell culture models[19]. As βS immunization and reduction has been shown in transgenic mice[20], down-regulation of βS expression in patients, either at the mRNA or protein level, may be technically possible in the near future.

## 5. Clinical evidence supporting the involvement of aEVO in OLs diseases

As described, up-regulated aEVO activity might reduce MS susceptibility in young adulthood, whereas MSA might occur later through antagonistic pleiotropy. Alternatively, if aEVO activity is decreased, then MS susceptibility might increase, while MSA risk might be reduced. Indeed, experimental evidence in the literature, both preclinical as well as clinical, might lend support to this concept.

### 5.1 MS

**Figure 2.**
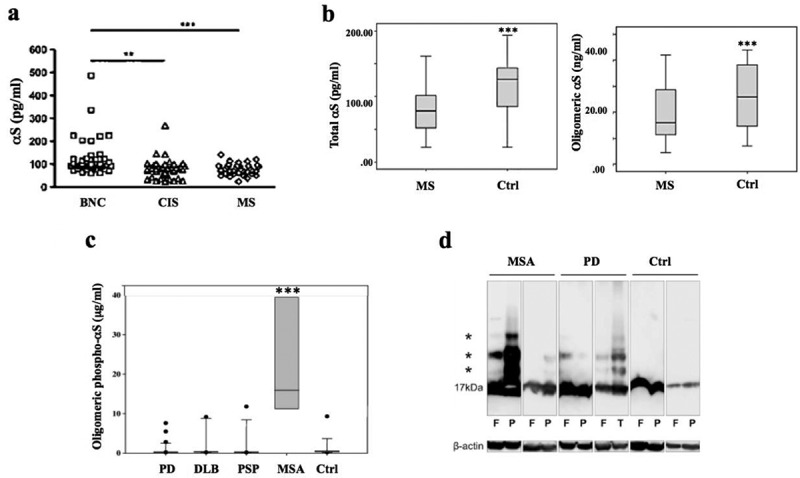
**Clinical evidence supporting altered αS evolvability in disorders with abnormal OL function**. (a), Levels of CSF αS in MS patients (*n* = 37) and in patients with clinically isolated syndrome (CIS, n = 36) were significantly decreased compared with those in benign neurological conditions (BNC, n = 38). **p< 0.01, ***p< 0.001. (b), Levels of αS and oligomeric αS in serum measured by ELISA were significantly lower in MS patients (*n* = 60) compared to the healthy control group (*n* = 60). ***p< 0.001. (c), Box-whisker plots revealed that oligomeric phosphorylated αS levels were elevated in post-mortem CSF derived from MSA (n = 8) and distinguished MSA from the other α-synucleinopathies, including PD (n = 38), DLB (n = 15), PSP (n = 12) and controls (n = 16), ***p< 0.001. (d), Detection of αS oligomers by immunoblotting. The sarkosyl-insoluble fractions of brain sample homogenates were analysed, and αS oligomers (asterisks) were visualized in fractions from MSA, PD and control (Ctrl) patients’ brain samples. F: frontal cortex, P: parietal cortex, T: temporal cortex. β-actin: loading control. Reprinted from Antonelou et al [21]. (a), Bilge et al [24]. (b), Foulds et al [22]. (c) and Sekiya et al [30]. (d) with permission.

Undoubtedly, CSF biomarkers have proven highly useful to investigate and monitor CNS disorders, including inflammatory and neurodegenerative disorders [21,22]. Notably, CSF βS was significantly decreased in both clinically isolated syndrome and in MS patients compared to controls (Figure 2a)[21]. Similarly, dermal βS expression level, as assessed by immunohistochemistry, was significantly reduced in relapsing-remitting MS compared to healthy controls[23]. Meanwhile, levels of serum monomeric and oligomeric βS were decreased in serum from MS patients (Figure 2b)[24]. In contrast, CSF αS levels were elevated in both MS and neuromyelitis optica patients reflecting worse disease disability[25]. Such discrepant findings parallel those in a well-established MS rodent model, namely experimental autoimmune encephalomyelitis triggered by myelin oligodendrocyte glycoprotein (MOG), in which both reduced αS expression[26], as well as increased levels are reported [27,28]. Specifically, in the former, significantly reduced neuroinflammation and βS were observed in βS knockout mice compared to wild-type animals[26], whereas, in the latter, αS expression increased in response to MOG treatment in rat without genetic manipulation[25]. Similar to βS, reduced Aβ expression in MS was also identified by brain PET[26], whereas Aβ precursor protein was rapidly induced in reactive glial cells and T cells in response to pathological stimuli including inflammation[29]. In our framework, these evidence might suggest that aEVO could involve a stress response in MS, including inflammation, but also appears to be reduced in the later chronic disease phase.

Also significant, βS-specific T cells were enriched in MS patients[16], suggesting that the balance between βS and βS might be critical for MS pathogenesis associated with T cell activation. Thus, reduced βS evolvability might result in increased inflammation, manifesting in MS pathology.

### 5.2 MSA

Conversely, markedly increased CSF βS in MSA distinguishes this condition from other α-synucleinopathies, including PD and DLB, and from progressive supranuclear palsy (PSP), a tauopathy (Figure 2c)[21]. Consistently, levels of βS monomers and oligomers in the sarkosyl-insoluble fractions of serum derived from MSA were increased compared with those from PD and healthy controls (Figure 2d)[30]. The reason why the CSF βS in MSA is higher than those in other α-synucleinopathies remains unclear, but it is possible that MSA βS strains differ from PD/DLB in their prion-like seeding capacity. Yet, a non-mutually exclusive alternative is that since OLs are specifically exposed to stressors for maintenance of myelin function, βS evolvability in OLs might be higher compared to those in other cell types. Of note, T cells from human MSA brain, and those from an MSA mouse model virally transduced with αS specific for oligodendrocytes, were reactive with αS peptides[31]. Thus, further investigations into the immune response by T cells in βS evolvability are warranted.

Furthermore, increased gene expression of amyloid precursor protein and other molecules related to Aβ production are demonstrated in MSA, suggesting that aEVO of APs, including βS and Aβ, might be up-regulated in MSA[32]. Altogether, these findings could support our notion that MS and MSA, the two forms of OL degeneration, might be linked through βS evolvability.

## 6. Conclusion

In our paper, we describe that AP protofibrils might be formed in parental brain in response to diverse stressors, such as AP aggregation and inflammation. Subsequently, AP protofibrils could be transmitted via germ cells in a prion-like fashion to confer stress information to offspring. Thus, aEVO may be analogous to the inheritance of acquired characteristics for environmental stressors. By virtue of aEVO, offspring can better cope with forthcoming stressors in the brain to reduce risk of MS. Increased aEVO in OLs, however, might result in MSA pathogenesis through the mechanism of antagonistic pleiotropy during ageing. Collectively, these two seemingly disparate disorders, MS and MSA, connected through OL pathology, might in fact be linked through aEVO.

Moreover, MS and MSA might be inversely related, the equilibrium of which is modulated by βS. Accordingly, reducing βS expression by ASO and/or immunotherapy might result in increased aEVO, which could be uniquely beneficial as an MS disease therapy strategy. Similarly, up-regulation of aEVO by reducing βS expression might be beneficial for various early degenerative diseases, including autosomal recessive PD and neuropathic types of lysosomal storage disorders[7]. On the other hand, since βS activity affects aEVO and correspondingly antagonistic pleiotropy in ageing, altered βS might be considered as a target of MSA and related disorders. On the whole, since βS might be involved in the development of both MS and MSA, this molecule could be an exciting therapeutic target for these conditions.

Much of our discussion in the current paper is based on theoretical considerations and definitely requires further supporting experimental evidence. Given that at least for MSA, no existing disease-modifying treatments are available, it is expected that our hypothesis of aEVO may lead to the development of therapeutic intervention for this condition. As for MS, this may identify alternate treatment strategies to supplement existing disease therapies, perhaps used in combination therapy. In summary, a clearer understanding of aEVO may lead to such new interventions for MA and MSA.
